# Schisandrin B promotes senescence of activated hepatic stellate cell via NCOA4-mediated ferritinophagy

**DOI:** 10.1080/13880209.2023.2189908

**Published:** 2023-04-03

**Authors:** Mingyue Ma, Na Wei, Jieren Yang, Tingting Ding, Anping Song, Lerong Chen, Shuguo Zheng, Huanhuan Jin

**Affiliations:** a Department of Pharmacology, School of Pharmacy, Wannan Medical College, Wuhu, Anhui, P.R. China; b Laboratory of Pharmacology of Chinese Medicine, School of Pharmacy, Wannan Medical College, Wuhu, Anhui, P.R. China

**Keywords:** Cellular senescence, nuclear receptor coactivator 4, hepatic fibrosis

## Abstract

**Context:**

Schisandrin B (Sch B), an active ingredient from *Schisandrae chinensis* (Turcz.) Baill. (Schisandraceae) Fructus, possesses diverse pharmacological activities including antitumor, anti-inflammation, and hepatoprotection.

**Objective:**

To explore the effect of Sch B on activated HSCs senescence in hepatic fibrosis and the mechanisms implicated.

**Materials and methods:**

ICR mice with CCl_4_-induced hepatic fibrosis were supplemented with Sch B (40 mg/kg) for 30 d and LX2 cells were treated with Sch B (5, 10 and 20 μM) for 24 h. Cellular senescence was assessed by senescence-related indicators senescence-associated β-galactosidase (SA-β-gal) activity and the expression of p16, p21, p53, γ-H2AX, H3K9me3, TERT, TRF1, and TRF2. Ferric ammonium citrate (FAC) and NCOA4 siRNA were used to evaluate the mechanisms underlying Sch B’s regulation of cellular senescence.

**Results:**

Sch B (40 mg/kg) reduced serum levels of AST and ALT (53.2% and 63.6%), alleviated hepatic collagen deposition, and promoted activated HSCs senescence in mice. Treatment with Sch B (20 μM) decreased cell viability to 80.38 ± 4.87% and elevated SA-β-gal activity, with the levels of p16, p21 and p53 increased by 4.5-, 2.9-, and 3.5-fold and the levels of TERT, TRF1 and TRF2 decreased by 2.4-, 2.7-, and 2.6-fold in LX2 cells. FAC (400 μM) enhanced Sch B’s effect mentioned above. NCOA4 siRNA weakened the effects of Sch B on iron deposition and HSCs senescence.

**Conclusions:**

Sch B could ameliorate hepatic fibrosis through the promotion of activated HSCs senescence, which might be attributed to its induction of NCOA4-mediated ferritinophagy and subsequent iron overload.

## Introduction

Hepatic fibrosis, a reversible wound-healing response triggered by a chronic liver injury, will eventually progress to liver cirrhosis, hepatocellular carcinoma and even liver failure if not treated properly (Schuppan and Kim 2013; Pellicoro et al. 2014; Forouzanfar et al. 2016). Hepatic fibrosis is mainly characterized by excessive deposition of extrahepatic matrix proteins and activation of hepatic stellate cells (HSCs). It is well known that activated HSCs are the principal cell type in the progression of liver fibrogenesis (Bataller and Brenner 2005) and intervention in the activation of HSCs has become an effective treatment measure. There have been a number of efforts to explore effective measures to control HSCs activation, including inhibiting HSCs proliferation, promoting HSCs apoptosis, autophagy or immune clearance (Zhang et al. 2014; Tsuchida and Friedman 2017). Recent studies revealed that induction of activated HSCs senescence was also an effective strategy for blocking the process of hepatic fibrosis (Krizhanovsky et al. 2008; Zhang et al. 2021). Cellular senescence, a response to different stresses, is a highly stable cell cycle arrest (Munoz-Espin and Serrano 2014), during which cells undergo some morphological, biochemical and functional changes including increased senescence-associated β-galactosidase (SA-β-gal) activity, DNA damage and the dysfunction of telomere and telomerase system (Bernadotte et al. 2016; Herranz and Gil 2018; Shmulevich and Shmulevic 2021).

Iron, an element necessary for various physiological activities, is involved in the formation of hemoglobin and some vital enzymatic groups. Iron homeostasis plays a key role in cellular and organism viability, and iron overload increases the risk of various diseases including liver disease (Haase et al. 2010). Growing evidence revealed that pathological iron overload could induce cellular senescence (Yang et al. 2017; Mangan 2021; Chen et al. 2022). Iron has a strong catalytic effect and generates reactive oxygen species (ROS) through the Fenton reaction, resulting in cellular oxidative damage and senescence (Dixon and Stockwell 2014; Nakamura et al. 2019; Qi et al. 2021; Yuan et al. 2021). Ferritinophagy, a cell-selective autophagy mediated by nuclear receptor coactivator 4 (NCOA4), is responsible for iron overload. NCOA4 can interact with ferritin heavy chain 1 (FTH1) in autophagosomes, leading to ferritin degradation and ferritin-bound iron release (Dowdle et al. 2014; Mancias et al. 2015). Increased intracellular free iron can then promote ROS accumulation and consequent cellular senescence (Qi et al. 2021).

Schisandrin B (Sch B), an active ingredient from *Schisandrae chinensis* (Turcz.) Baill. (Schisandraceae) Fructus, has been shown to possess diverse pharmacological effects, such as antitumor, antioxidant, anti-inflammation, and hepatoprotection (Leong and Ko 2016; Zhang et al. 2019; Cuiqiong et al. 2020; Chen et al. 2021; Yang et al. 2022). Recent studies showed that Sch B could inhibit hepatic fibrosis by regulating of HSCs activation, proliferation, and apoptosis (Chen et al. 2017; Cuiqiong et al. 2020). However, there is no relevant report on whether Sch B can regulate the senescence of activated HSCs. In this study, we investigated the ameliorative effect of Sch B on HSCs senescence and the underlying mechanisms from the aspect of NCOA4-mediated ferritinophagy.

## Materials and methods

### Drugs and chemicals

Sch B(CAS number: 61281-37-6, Herbpurify, Chengdu, China); Ferric ammonium citrate (FAC) (Solarbio, Beijing, China); Lipofectamine 2000 reagent (Invitrogen, Thermo Fisher Scientific, Waltham, MA, USA); SA-β-gal staining kit (Cell Signaling Technology, Danvers, MA, USA); Cell counting kit-8 (CCK-8), 5-ethynyl-2′-deoxyuridine (EdU) cell proliferation kit, BCA protein assay kit (Beyotime Biotechnology, Shanghai, China); Iron colorimetric assay kit (APPLYGEN, Beijing, China); Prussian blue staining kit (LEAGENE, Beijing, China); ROS assay kit (Yeasen Biotechnology, Shanghai, China); DMEM (Gibco, Grand Island, NY, USA); Fetal bovine serum (ExCell Bio, Shanghai, China); Pierce Co-Immunoprecipitation (Co-IP) kit (Thermo Fisher Scientific, Waltham, MA, USA); Alanine aminotransferase (ALT), Aspartate transaminase (AST) assay kits (Nanjing Jiancheng Bioengineering Institute, Nanjing, China). The following antibodies were used in this study: p16, p21, p53, H3K9me3, γ-H2AX, LC3B (Cell Signaling Technology Danvers, MA, USA); FTH1, α-SMA, TRF1 (Santa Cruz Biotechnology, Santa Cruz, CA, USA); TRF2, β-Actin (ProteinTech Group, Chicago, IL, USA); TERT (Bioss, Beijing, China).

### Experimental animal procedures

Male ICR mice (6–8 weeks, 20–30 g) were bought from Changsha Tianqin Biotechnology Co., Ltd. All animal experimental procedures were approved by the Animal Care and Use Institution and Local Committee of Wannan Medical College (No. LLSC-2020-141). Mice were housed under a 12 h light/dark cycle and maintained at constant temperature (21 ± 2 °C) and humidity (45 ± 10%) at Laboratory Animal Research Center. Following adaptive feeding on a standard diet for one week, mice were randomly divided into 3 groups (*n* = 10). Mice in group 2 and group 3 were injected intraperitoneally with CCl_4_ (diluted at 1:5 (v/v) in olive oil, 5 µL/g body weight) once every three days for 60 days to induce hepatic fibrosis, while those in group 1 received intraperitoneal injection of olive oil. Mice in group 3 were administered intragastrically with Sch B (40 mg/kg/d) from the 31st day to the end of the experiment. Following anesthetizing with an intraperitoneal injection of pentobarbital, blood was collected and the animals were sacrificed, and part of the liver tissue was fixed with 10% formalin and the rest was stored at −80 °C for future experiments.

### Serum biochemical analysis

Serum levels of liver injury indexes (ALT and AST) were detected by commercial assay kits according to the manufacturer’s protocol.

### Hematoxylin and eosin and Masson staining

Liver tissues were fixed in formalin solution and then embedded in paraffin. Four µm thickness paraffin sections were stained with hematoxylin and eosin or with Masson’s trichrome to determine the degree of liver injury and collagen distribution.

### Cell culture

Human hepatic stellate cell line LX2 was purchased from Shanghai Fuheng Biotechnology Co., Ltd. **(**Shanghai, China). LX2 cells were grown in dulbecco’s modified eagle’s medium (DMEM) supplemented with 10% fetal bovine serum (FBS), 1% penicillin and 1% streptomycin. LX2 cells were grown at 37 °C in a wet incubator containing 5% CO_2_.

### Cell viability assay

LX2 cells cultured in 96 well plates were incubated with Sch B at indicated concentrations for 24 h. Cell viability was evaluated using CCK-8 kit according to the manufacturer’s instructions. The absorbance was then measured using a microplate reader (Infinite 200PRO, Tecan, Männedorf, Switzerland) at 450 nm.

### EdU incorporation assay

Cell proliferation was detected according to the manufacturer’s instructions for the BeyoClick EdU-488 kit. Cells were seeded in 24-well flat-bottom microtiter plates at a density of 20,000 cells per well. Following treatment with different concentrations of Sch B for 24 h, LX2 cells were incubated with the fresh DMEM and EdU staining buffer at 37 °C for 2 h. The cells were then fixed with 4% paraformaldehyde at 20 °C for 30 min before staining with Hoechst for 3 min. Positive cells were observed under an inverted fluorescence microscope (Version 6.1, Zeiss, Oberkochen, Germany) and fluorescence intensity was analyzed quantitatively with Image J software.

### Immunofluorescence staining

Frozen liver tissue was embedded in an optimal cutting temperature (OCT) compound and cut into 8 μm sections (Jin et al. 2016). The liver tissues and cells were incubated overnight at 4 °C with the primary antibodies against H3K9me3, α-SMA and p21 (1:200), followed by washing with PBS 3 times and incubation with secondary antibodies (ProteinTech Group, Chicago, IL, USA) at room temperature for 2 h. 4'6-diamidino-2-phenylindole (DAPI) solution was added and incubated for 15 min. Images were captured with laser confocal microscopy (Leica TCS SP8, Leica, Wetzlar, Germany).

### Fe^2+^ release assay

Intracellular iron content is measured by the Iron colorimetric assay kit. LX2 cells cultured in 96 well plates were incubated with Sch B and other reagents as described in Figure 3(b) and 5(b). Iron reducer solution (50 μL) was added into samples and incubated at 60 °C for 60 min. Next, 15 μL iron probe solution was added into samples and incubated at 25 °C for 30 min. The absorbance at 550 nm was measured using a microplate reader.

### Prussian blue staining

Prussian blue staining kit was used to detect iron content in cells and tissues according to the manufacturer’s instructions. LX2 cells cultured in 24 well plates were incubated with Sch B as described in Figures 3(c) and 5(c). LX2 cells were fixed with 4% paraformaldehyde for 20 min and incubated with Prussian blue staining solution at 37 °C for 3 h. The blue particles were observed under an inverted microscope. Frozen sections of mice livers were stained as mentioned above.

### Cell transfection with si-NCOA4

The si-NCOA4 (Tsingke Biotechnology, Beijing, China) and blank vector were transiently transfected into LX2 cells by lipofectamine 2000. Cells were incubated with a transfection complex solution (containing 1 μg si-NCOA4 or negative siRNA and 4 μL lipofectamine 2000) at 37 °C for 7 h. Then, the cells were incubated with fresh medium at 37 °C for 17 h. The sequence of si-NCOA4 (human): 5'–-GACCUUAUUUAUCAGCUUA–3'.

### SA-β-gal staining

**Figure 1.**
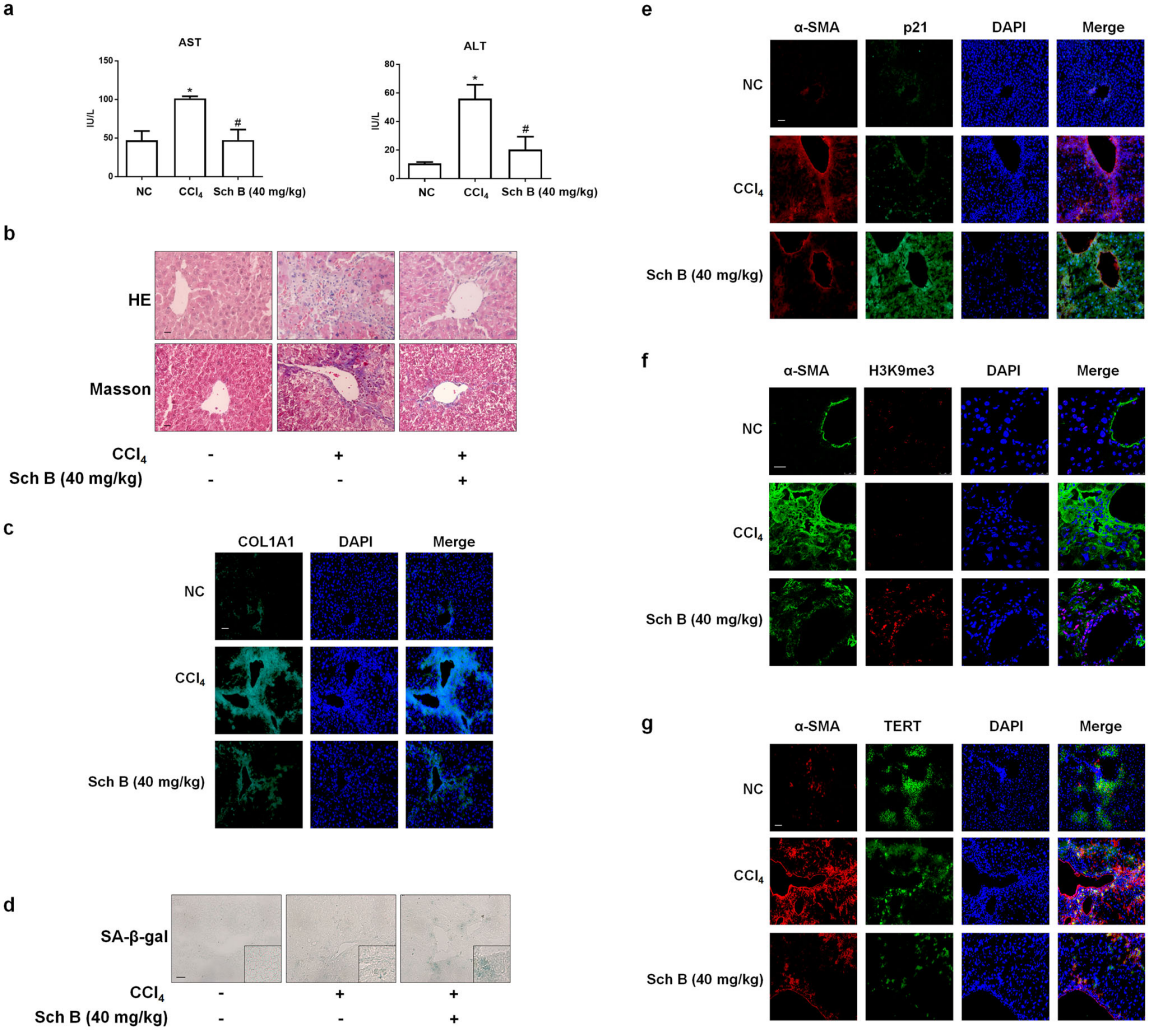
Sch Bameliorated hepatic fibrosis through modulating the senescence of activated HSCs. Male ICR mice were grouped as follows: group 1, vehicle control (no CCl_4_, no treatment); group 2, model group (with CCl_4_, no treatment); CCl_4_ + Sch B-treated group (40 mg/kg). (a) Determination of serum ALT and AST levels. Data were expressed as mean ± SD. Significance: *P < 0.05 vs. Control, *^#^P* < 0.05 vs. CCl_4_ group (*n* = 3). (b) H&E and Masson staining of liver sections. Scale bars, 20 μm (*n* = 3). (c) Immunofluorescence analyses of COL1A1 in liver tissues (n = 3). DAPI was used to stain the nucleus. Scale bar, 50 μm. (d) SA-β-gal staining of liver sections. Scale bars, 50 μm (*n* = 3). (e, f, g) Immunofluorescence analyses of p21, H3K9me3 (Scale bar, 25 μm) and TERT (Scale bar, 50 μm) in liver tissues (n = 3). Staining with α-SMA was used to identify activated HSCs. DAPI was used to stain the nucleus.

**Figure 2.**
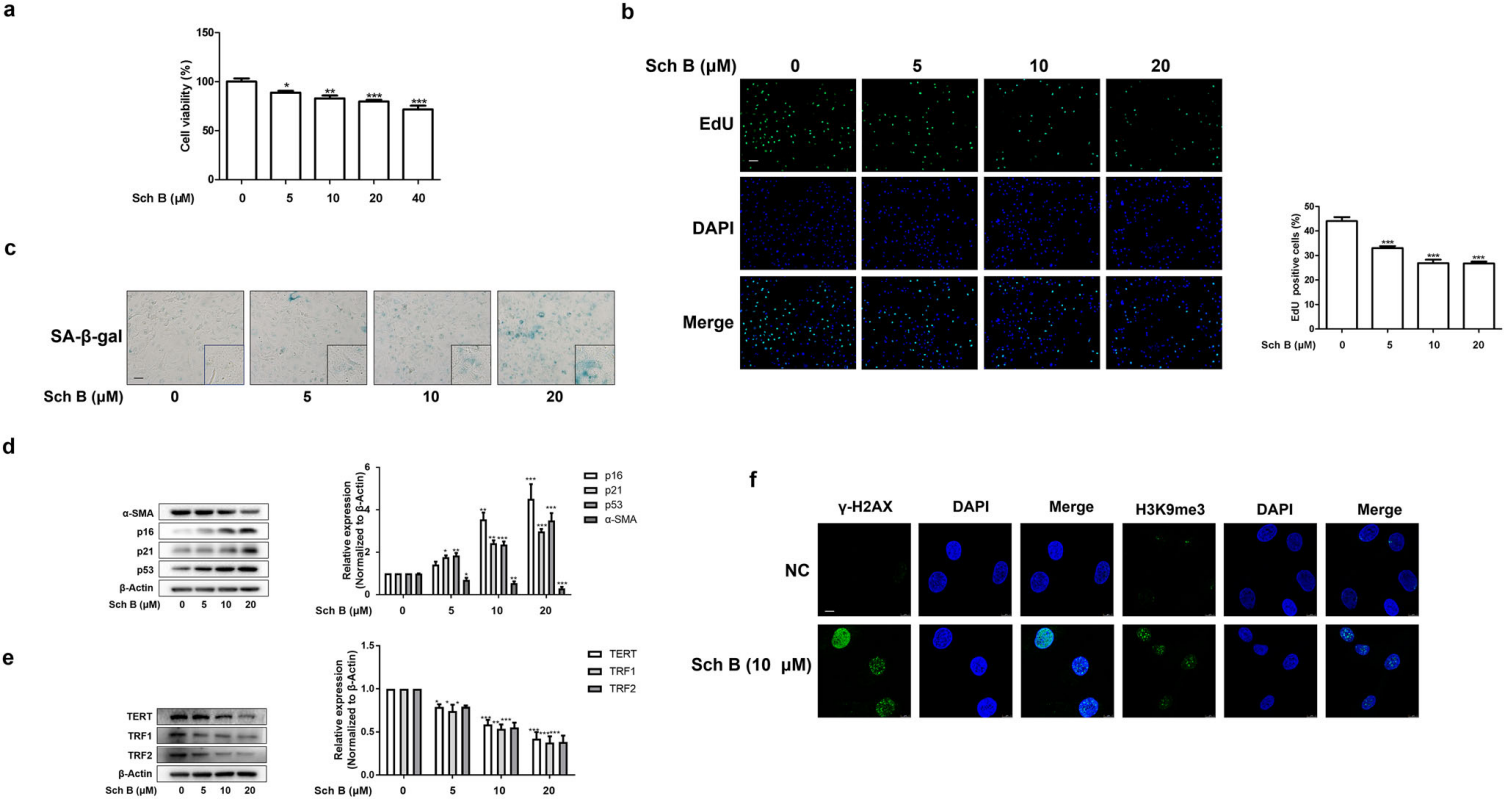
Sch B promoted the senescence of activated HSCs *in vitro*. When LX2 cells grew to 70–80% confluence, cells were incubated with different concentrations of Sch B for 24 h. (a) CCK-8 analysis of cell viability, *n* = 5. Data were expressed as mean ± SD. Significance: *P < 0.05,**P < 0.01, ***P < 0.001 vs. Control. (b) EdU incorporation assay in LX2 cells. Scale bar, 100 μm (*n* = 3). Data were expressed as mean ± SD. Significance: ***P < 0.001 vs. Control. (c) Representative images of SA-β-gal staining in LX2 cells. Scale bar, 50 μm (*n* = 3). (d, e) Western blot analyses of indicators of HSCs activation (α-SMA), senescence markers (p16, p21 and p53) and telomere/telomerase-related factors (TERT, TRF1 and TRF2) in LX2 cells (*n* = 3). Data were expressed as mean ± SD. Significance: *P < 0.05, **P < 0.01, ***P < 0.001 vs. Control. (f) Immunofluorescence analysis of H3K9me3 and γ-H2AX expression. Nuclear was showed by DAPI. Scale bar, 10 μm (*n* = 3).

**Figure 3.**
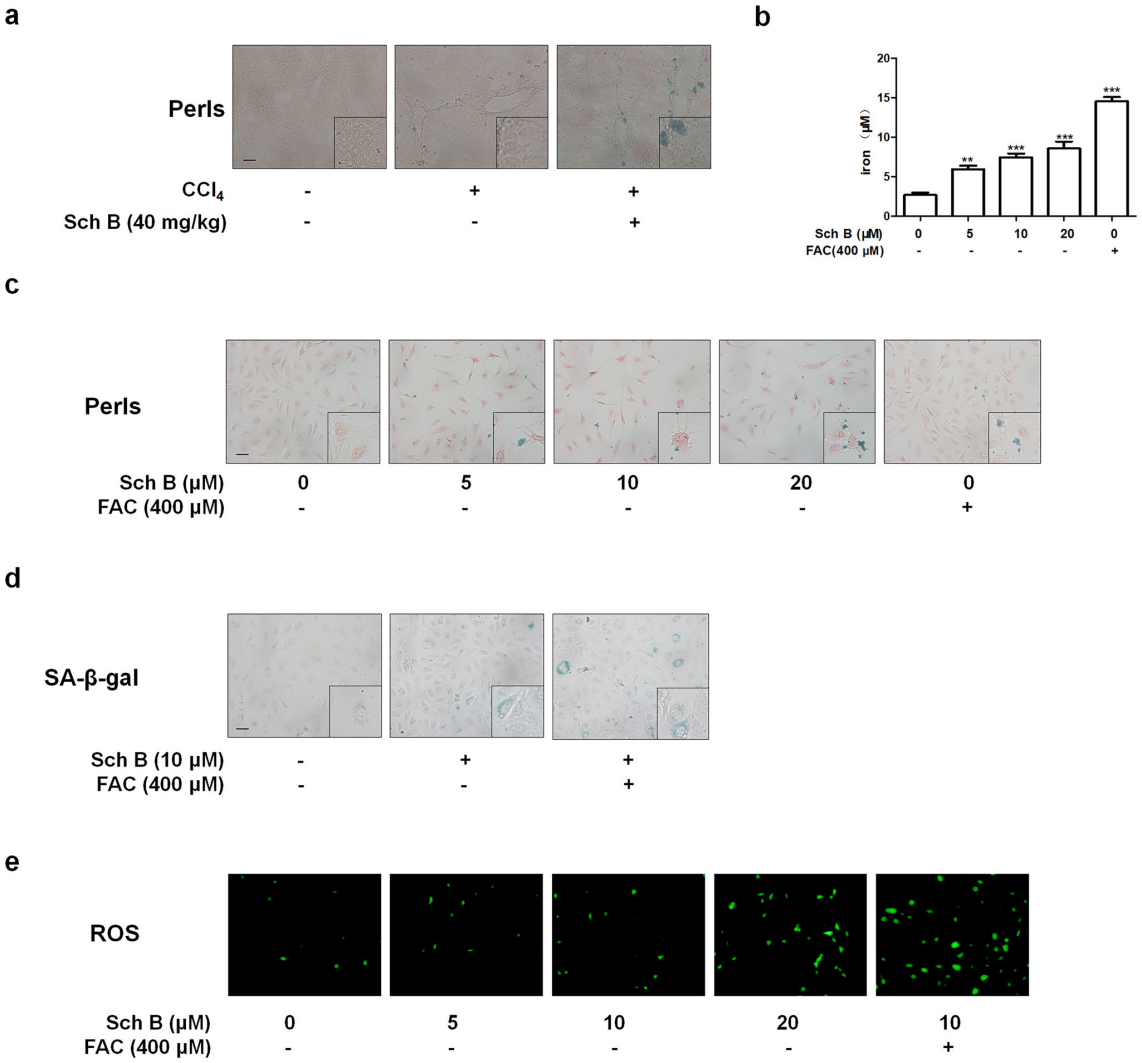
Sch B promoted the senescence of activated HSCs through induction of iron overload. (a) Liver sections stained with Perls reagents for iron deposition examination. Scale bars, 50 μm (*n* = 3). (b) Iron colorimetric assay of iron content in LX2 cells (*n* = 4). Data were expressed as mean ± SD. Significance: **P < 0.01, ***P < 0.001 vs. Control. (c) Prussian blue staining analysis of iron content in LX2 cells. Scale bar, 50 μm (*n* = 3). (d) SA-β-gal analysis of the senescence of LX2 cells. Data were presented as a percentage of SA-β-gal-positive cells per unit area. Scale bar, 50 μm (*n* = 3). (e) Determination of ROS content in LX2 cells by DCFH-DA labeling. Scale bar, 50 μm (*n* = 3).

The activity of β-galactosidase in cells and tissues was detected using the SA-β-gal kit according to the manufacturer’s instructions. LX2 cells cultured in 24 well plates were incubated with Sch B and other reagents as described in Figures 1(d), 2(c), 3(d) and 5(e), and fixed with a fixative solution for 15 min. The SA-β-gal staining solution with pH value of 5.9 ± 0.2 was added to a 24-well plate (500 μL/well) and incubated in a drying oven at 37 °C for 12 h. Frozen sections of mice livers were stained as mentioned above.

### Intracellular ROS assay

Intracellular ROS was detected by 2',7'-dichlorodihydrofluorescein diacetate (DCFH-DA). LX2 cells cultured in 6 well plates were incubated with Sch B and other reagents as described in Figures 3(e) and 5(d). Cells were incubated with 5 μM DCFH-DA for 30 min at 37 °C. Images were captured from three randomly selected fields using an inverted fluorescence microscope.

### Co-immunoprecipitation assay

The immunoprecipitation method is consistent with previous research methods (Qi et al. 2021). The AminoLink coupling resin bound to the FTH1 antibody and the negative control IgG antibody was fixed on the AminoLink coupling resin, respectively. Protein concentration was determined using the BCA kit. The protein supernatant was added to the resin column and filtered to collect the eluent. NCOA4 binding to FTH1 was observed using the western blotting method.

### Western blot assay

Protein concentrations of cell lysates were determined using BCA protein assay kit. The western blotting analysis was performed as previously described (Jin et al. 2017). The protein bands were visualized using ECL detection kit (Millipore, MA, USA). The levels of target protein bands were determined by Image Lab or Image J and normalized to that of β-actin.

### Statistical analysis

Data were presented as mean ± SD and analyzed using GraphPad software (GraphPad 5, San Diego, CA, USA). The significance of the difference was determined *via* a one-way analysis of variance with the *post hoc* Dunnett’s test. Values of *p*** **<** **0.05 were considered statistically significant.

## Results

### Sch B ameliorated hepatic fibrosis through modulating the senescence of activated HSCs

We initially evaluated the effect of Sch B on CCl_4_-induced liver fibrosis in mice. The results showed that treatment with Sch B alleviated CCl_4_-induced liver injury as exhibited by increased serum levels of ALT, AST, disorganized hepatocytes, loss of hepatic lobule structure and liver collagen deposition (Figure 1(a–c)). The results of SA-β-gal staining showed that Sch B treatment increased HSCs senescence in CCl_4_-treated mice liver (Figure 1(d)). In addition, Sch B treatment increased the expression of p21 (senescence-related indicator), H3K9me3 (DNA damage index) and decreased the expression of TERT (telomerase system-related index) in the activated HSCs (α-SMA as the key marker of HSC activation) of the fibrotic liver (Figure 1(e–g)). Altogether, these data indicated that the promotion of activated HSCs senescence by Sch B might contribute to its improvement of CCl_4_-caused liver fibrosis *in vivo*.

To provide further evidence for the senescence-inducing effect of Sch B in activated HSCs, we examined the effect of Sch B in cultured human HSC line-LX2 cells. Treatment with different concentrations of Sch B for 24 h decreased the viability of LX2 cells (Figure 2(a)). Therefore, we used doses of 5, 10, 20 μM Sch B for the following experiments. The proportion of EdU-positive cells was significantly reduced by Sch B treatment (Figure 2(b)). Moreover, the activity of SA-β-gal was elevated by Sch B (Figure 2(c)). Furthermore, we examined the protein abundance of α-SMA and senescence-related indicators such as p16, p21 and p53. The results showed that Sch B decreased the expression of α-SMA and increased the expression of senescence-related indicators (Figure 2(d)). In addition, Sch B reduced the expressions of TERT, TRF1 and TRF2 *in vitro* (Figure 2(e)). Consistently, immunofluorescence staining showed that the levels of DNA damage index γ-H2AX and H3K9me3 (Oyama et al. 2018) were elevated by Sch B treatment (Figure 2(f)). Collectively, Sch B could promote the senescence of activated HSCs *in vivo* and *in vitro*.

### Sch B promoted the senescence of activated HSCs through induction of iron overload

We next explored the role of iron overload in Sch B regulation of cellular senescence. Prussian blue staining showed that Sch B dramatically exacerbated iron deposition in both *in vivo* and *in vitro* models of liver fibrosis (Figure 3(a–c)). We used iron overload inducer FAC to identify whether iron overload was involved in Sch B regulation of HSCs senescence. Sch B at 10 μM and FAC at 400 μM significantly accelerated cellular senescence in LX2 cells (Figure 3(d)). Furthermore, Sch B increased intracellular ROS levels and FAC enhanced the effect of Sch B on ROS level in LX2 cells (Figure 3(e)). Taken together, these data indicated that Sch B could promote iron accumulation and subsequent oxidative stress, which might play a central role in mediating the senescence of activated HSCs.

### Sch B regulated NCOA4-mediated ferritinophagy in activated HSCs

**Figure 4.**
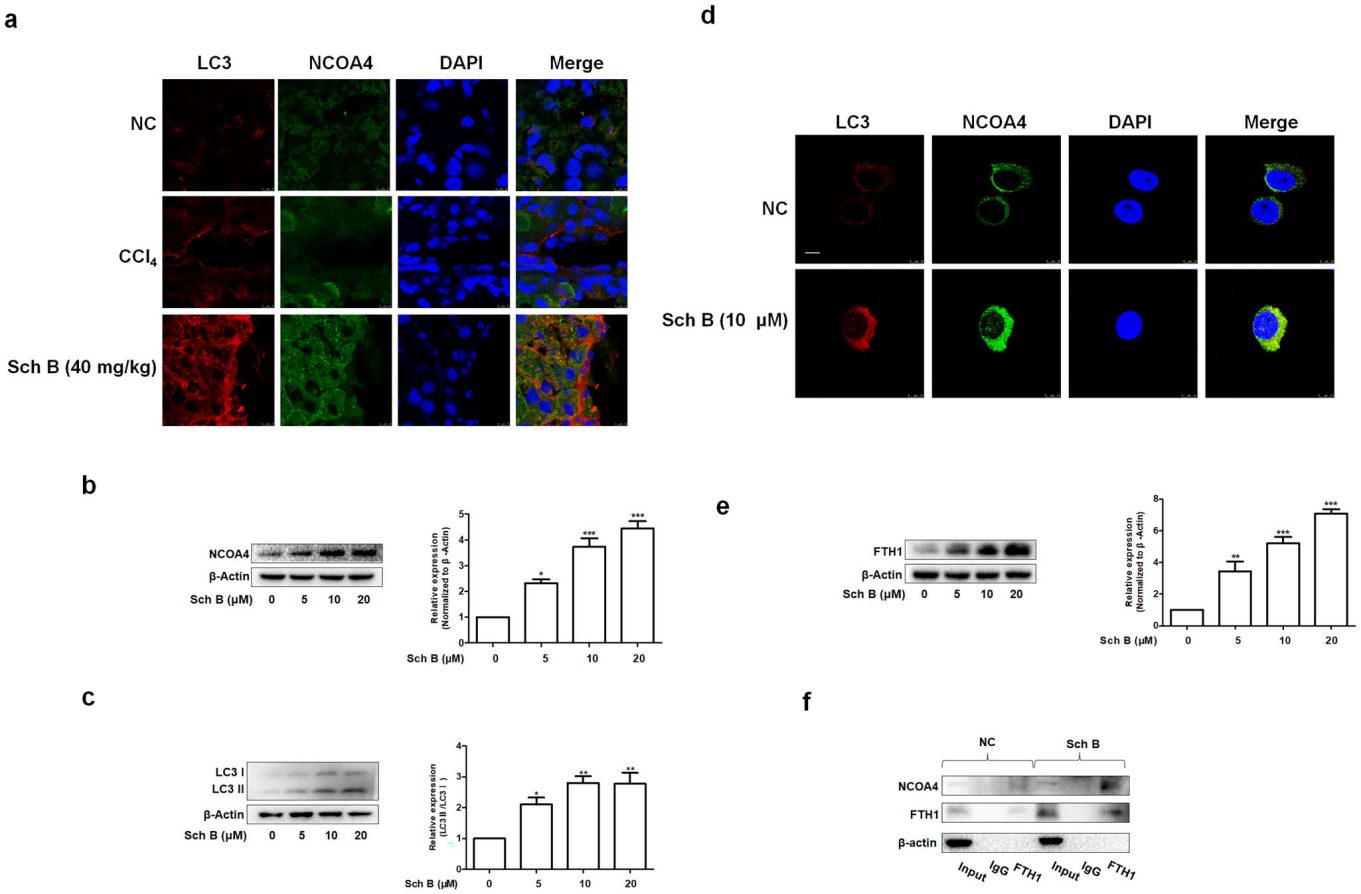
Sch B regulates NCOA4-mediated ferritinophagy in activated HSCs. (a) Immunofluorescence staining analysis of the localization of NCOA4 and LC3B in liver tissue. Nuclear was shown by DAPI. Scale bar, 10 μm (*n* = 3). (b, c) Western blot analyses of NCOA4 and LC3B in LX2 cells (*n* = 3). Data were expressed as mean ± SD. Significance: *P < 0.05, **P < 0.01, ***P < 0.001 vs. Control. (d) Immunofluorescence staining analysis of the localization of NCOA4 and LC3B in LX2 cells. Nuclear was shown by DAPI. Scale bar, 7.5 μm (*n* = 3). (e) Western blot analyses of FTH1 in LX2 cells (*n* = 3). Data were expressed as mean ± SD. Significance: **P < 0.01, ***P < 0.001 vs. Control. (f) Co-IP analysis of the interaction between FTH1 and NCOA4.

We next examined the mechanisms underlying Sch B’s regulation of activated HSCs senescence *via* iron overload. The analyses of immunofluorescence and western blot indicated that Sch B up-regulated the protein expression of microtubule-associated protein 1 light chain 3β (LC3B) and NCOA4 in the fibrotic mice liver as well as cultured LX2 cells (Figure 4(a–-d)). More importantly, Sch B treatment made NCOA4 accumulate mainly in autophagosomes, of which LC3B was used as a classic autophagosome marker (Figure 4(a,b)). To further examine whether iron overload is caused by ferritinophagy, we assessed the effect of Sch B on the expression of FTH1 in cultured LX2 cells. The protein expression of FTH1 was increased by Sch B (Figure 4(e)). Additionally, Sch B promoted the binding of NCOA4 to FTH1 *in vitro* (Figure 4(f)). Collectively, these data suggested that Sch B could induce iron overload by ferritinophagy in hepatic fibrosis.

### Inhibition of ferritinophagy weakened the effects of Sch B on iron overload and cellular senescence in activated HSCs

**Figure 5.**
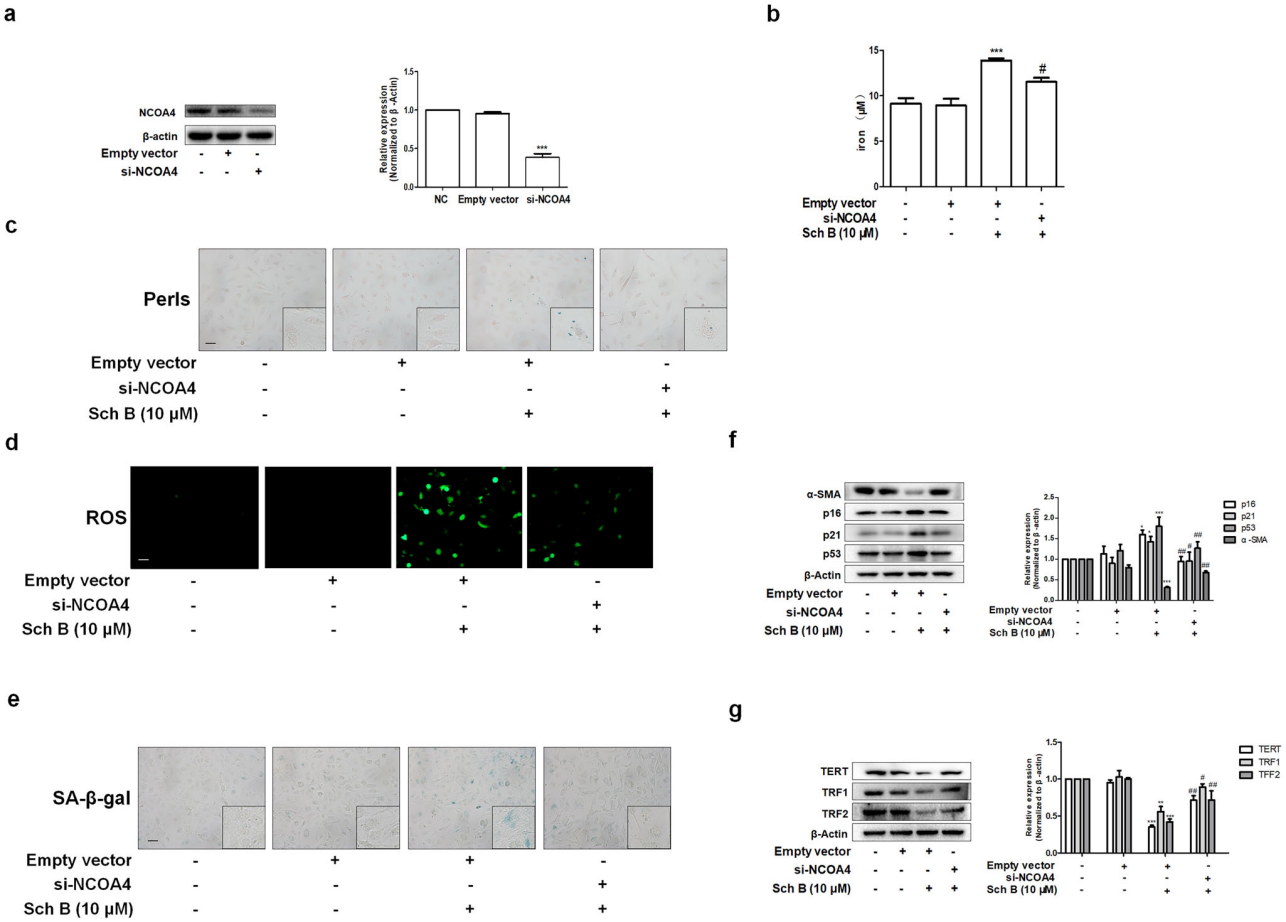
Inhibition of ferritinophagy weakened the effects of Sch B on iron overload and cellular senescence in activated HSCs. (a) The transfection efficiency of NCOA4 siRNA measured by western blot analysis. Data were expressed as mean ± SD. Significance: ***P < 0.001 vs. Empty vector. (b) Iron colorimetric assay in LX2 cells (*n* = 4). Data were expressed as mean ± SD. Significance: ***P < 0.001 vs. Empty vector, ^#^*P* < 0.05 vs. Empty vector + Sch B. (c) Prussian blue staining analysis of iron content in LX2 cells. Scale bar, 50 μm (*n* = 3). (d) Determination of ROS content in LX2 cells by DCFH-DA labeling. Scale bar, 50 μm (*n* = 3). (e) SA-β-Gal staining analysis in LX2 cells. Scale bar, 50 μm (*n* = 3). (f, g) Western blot analyses of HSCs activation (α-SMA), senescence markers (p16, p21 and p53) and telomere and telomerase related factors (TERT, TRF1 and TRF2) in LX2 cells (*n* = 3). Data were expressed as mean ± SD. Significance: *P < 0.05, **P < 0.01, ***P < 0.001 vs. Empty vector, ^#^*P* < 0.05, ^##^*P* < 0.01 vs. Empty vector + Sch B.

In order to further test whether Sch B induced iron overload and cellular senescence through activation of ferritinophagy, we transfected LX2 cells with NCOA4 siRNA and the transfection efficiency was validated using western blotting (Figure 5(a)). Firstly, si-NCOA4 significantly counteracted the effect of Sch B on iron deposition in LX2 cells (Figure 5(b,c)). Moreover, Sch B’s effects on intracellular ROS were also offset by transfection with si-NCOA4 (Figure 5(d)). Furthermore, Sch B-stimulated cellular senescence in activated HSCs was abolished by NCOA4 siRNA transfection (Figure 5e). Similar results were obtained by detecting the senescence markers and telomerase system indices (Figure 5(f,g)). Overall, these data further support the idea that Sch B could promote cellular senescence by regulating ferritinophagy-mediated iron overload in HSCs.

## Discussion

It is well known that about half of all disease deaths in developed countries are closely associated with chronic fibroproliferative diseases, including hepatic fibrosis (Kong et al. 2019). HSCs activation correlated closely with the development of hepatic fibrosis and the promotion of activated HSCs senescence has been recognized as an important method for the prevention and treatment of hepatic fibrosis (Krizhanovsky et al. 2008; Zhang et al. 2021). Cellular senescence is characterized by decreased proliferation capacity, increased SA-β-gal activity, senescence-related protein expression and the dysfunction of the telomere and telomerase system (Dang et al. 2020; Di Micco et al. 2021; Wang et al. 2022). TERT, a key telomerase for cell survival, prevents telomere shortening and subsequent cellular senescence in the cells undergoing multiple divisions (Nault et al. 2019; Azarm et al. 2020). TRF1 and TRF2 are involved in normal telomere replication and T-ring formation, respectively (Coluzzi et al. 2019; El Mai et al. 2021). Studies over the past decade have implicated the pivotal role of TRF1 and TRF2 in the process of cellular senescence (Coluzzi et al. 2019; Azarm et al. 2020). Although Sch B could inhibit hepatic fibrosis in rats (Leong and Ko 2016; Zhang et al. 2019; Cuiqiong et al. 2020; Chen et al. 2021), there are no in-depth reports focusing on the effect of Sch B on HSCs senescence in hepatic fibrosis. Elucidation of the mechanisms governing the senescence of activated HSCs may provide a therapeutic approach for Sch B to control liver fibrosis. In the current study, we initially demonstrated that Sch B promoted the senescence of activated HSCs in both *in vivo* and *in vitro* models of liver fibrosis, as manifested by the major hallmarks of cellular senescence including SA-β-gal activity, the expression of p16, p21, p53, H3K9me3, γ-H2AX and telomere/telomerase system. Thus, it is conceivable that Sch B’s inhibition of hepatic fibrosis could be related to its promotion of activated HSCs senescence.

It is well known that iron homeostasis is essential for cellular and organism viability, and its imbalance can lead to metabolic disorders and various diseases (Fernandez-Real and Manco 2014; Bogdan et al. 2016). The liver is an important iron storage organ and excessive accumulation of iron is conducive to the production of ROS through the Fenton reaction (Nakamura et al. 2019; Halcrow et al. 2021; Qi et al. 2021), which has been implicated in the induction of premature senescence response (Roy et al. 2012; Ameziane-El-Hassani and Dupuy 2017). We further investigated the mechanisms underlying Sch B’s regulation of activated HSCs senescence and identified a key role for iron overload in this context. Sch B could increase the intracellular iron content and ROS level in LX2 cells, and these effects could be enhanced by an iron overload inducer FAC, which could further aggravate the senescence of activated HSCs. In this part of the results, it is better to use an iron overload inhibitor rather than an iron overload inducer to illustrate that Sch B affects cellular senescence by inducing iron overload, but there is no ideal iron overload inhibitor at present. Nevertheless, based on previous reports that iron overload could induce cellular senescence, it is reasonable to deduce that iron overload-induced ROS production could be involved in Sch B’s induction of cellular senescence in activated HSCs.

Ferritinophagy can be categorized into two parts: the formation of autophagosomes and the targeted recognition of ferritin (Xiu et al. 2022). NCOA4 is a selective cargo receptor that mediates the autophagic degradation of iron proteins in the cytoplasmic iron storage complex (Mancias et al. 2015; Santana-Codina and Mancias 2018; Kong et al. 2019; Li et al. 2020). In the current study, Sch B elevated the expression of LC3B and NCOA4 and promoted protein-protein interaction between NCOA4 and FTH1. These results indicated that the promotion of iron overload by Sch B might be due to its induction of ferritinophagy. Subsequently, si-NCOA4 was used to investigate the role of ferritinophagy on Sch B’s regulation of iron overload and cellular senescence. The results indicated that si-NCOA4 could reverse Sch B’s induction of iron overload, ROS production and cellular senescence in LX2 cells. These data provided further evidence for the notion that Sch B’s induction of cellular senescence might be derived from its activation of ferrtinophagy in HSCs.

## Conclusions

The aggregate data in this study indicated that Sch B regulated iron overload-mediated cellular senescence through ferritinophagy in activated HSCs, indicating that selective promotion of ferritinophagy may represent a therapeutic target for induction of activated HSCs senescence by Sch B. Overall, our study provided a novel molecular basis for the development of Sch B as a promising anti-hepatic fibrosis drug.

## Data Availability

The data that support the findings of this study are available from the corresponding author upon reasonable request.
